# Progress in Surgery

**Published:** 1898-12-17

**Authors:** 


### Progress in Surgery.

### TUMOURS.

**Subserous Sarcoma of the Peritoneum.**—Dr. O. T. Kauffmann[1] reports a case of this kind in a female child aged four. The case ran a rapid course, beginning with loss of flesh and appetite ten weeks before death, and only having abdominal swelling during the last month of life. When examined, a fortnight before death, the abdomen was found to be uniformly enlarged, semi-elastic, painless, with flattened umbilicus, but no bulging in the flanks. The sides were dull, the centre resonant over a narrow oval area. A swelling could be felt in the right inguinal and lumbar regions. The condition was thought to be one of tuberculous peritonitis, with glandular enlargement in the inguinal region. A few ounces of fluid were removed from the abdomen by paracentesis. The child rapidly sank. The post-mortem showed diffuse subserous thickening, affecting both the parietal and visceral peritoneum. There were no adhesions. The recti-abdominis and diaphragm were invaded. The large intestine was enormously enlarged and the small intestine considerably throughout, and also the mesentery and omentum. All the mucous membrane was healthy. Each kidney and the right pleura contained secondary nodules. The neoplasm proved to be a round-celled sarcoma.

**Retro-Peritoneal Fibro-Myoma Invading Mesentery.**—Binaud and Begouin[2] report a case of this affection. The patient was a woman of 50 and had had the tumour one year. It was well defined, hard, somewhat movable in all directions, and extended from the hypogastrium to within a finger's breadth of the umbilicus, and from the right superior anterior spine of the ilium to two inches to the left of the median line. The uterus was small, retroverted, and entirely free from the tumour, which extended into the right fornix. Laparotomy was performed. A coil of small intestines and the cæcum were found stretched out on the tumour. The uterus and ovaries were quite distinct from the growth which had opened up the mesentery. The patient did well for a fortnight and then became suddenly ill and died on the seventeenth day. Two drachms of pus were found in the cavity from which the tumour had been removed. Marmaduke Shield reported a similar case in 1897 in the Medico-Chirurgical Transactions.

**Metastatic Carcinoma of Choroid.**—Legrange[2] (Rue d'Ophtal, 1897) summarises 18 recorded cases of this affection, and adds a case of his own. The left breast had been removed four years previously for cancer. The right eye became painful and blind. A new growth was visible in the outer quadrant of the iris, bossy and sharply defined. The eye was excised. Later in the year signs of cancer of the spinal column showed themselves. The relative rarity of ocular metastases is explained by the narrow calibre of the ophthalmic artery, and by its being set at right angles to the internal carotid artery. It may affect one or both eyes, and the left eye is the most often affected.

**Sarcoma of the Œsophagus.**—Dr. L. E. Livingood[3] records a case of the disease in a man, æt. 55. The symptoms began with dysphagia seven months before the fatal ending. The pain was at the sternal notch and not at first constant. It was increased by swallowing. Three months later a tube was passed into the stomach. Its passage was slightly obstructed just before entering the stomach, and a little blood was found in the end. As the case progressed difficulty in swallowing liquids became marked. Once he coughed up a fragment that looked like necrotic tissue and blood, but it was too decomposed to identify microscopically. For several days after this swallowing was easier. Four days before death he was seized with very severe pain over the lumbar region, and between the shoulders. He soon sank into a state of collapse after this. The post-mortem revealed the new growth to be made up of a main mass, with several smaller polyp-like masses extending downwards from it. The edge of the main mass was a thick circular ridge; the central part was crater-like and necrotic, and communicated by a short fistulous channel with a gangrenous cavity in the right lung. The tumour masses were firm, elastic, and not friable, except where necrotic. It had, macroscopically, a shining, uniform, white, or slightly pink appearance in section. It did not infiltrate the œsophageal walls at its periphery as a carcinoma would. Microscopically, it was composed of spindle cells of varying sizes, often arranged in whorls, but with no alveolar appearance. The symptoms of this and the few other cases on record, of which four are alluded to by the writer, are the same as those of the ordinary carcinoma. Metastases are infrequent as in carcinoma. Probably the apparent rarity of this lesion is due to lack of careful histological study. The growth seems to begin in the mucous or submucous layer of the œsophageal wall.

**Interscapulo-thoracic Amputation for Sarcoma.**—Mr. Barling[4] recently detailed two cases in which he had performed this operation for sarcoma of the humerus. The first case was of a man aged 53, who had had the tumour three and a half years. The growth was diagnosed as a myeloid sarcoma. It occupied the upper two-thirds of the arm and overlapped the shoulder-joint, the outer end of the clavicle, and the spine of the scapula, filled the axilla, and stretched under the pectoral muscles. The operation was a slight modification of Berger's. Recovery was good, but six months later there were many secondary growths. The second case was diagnosed as a periosteal sarcoma; it occupied the upper two-thirds of the arm and overlapped the shoulder-joint. There was no recurrence fifteen months after the operation. A collection of 19 cases recorded since 1890 showed that all the patients recovered, indicating that the mortality was not 21 per cent., as previously estimated. One of the difficulties of the operation is to secure the vein without making a hole into it. Mr. Boyd suggests that more than the middle third of the clavicle should be removed, as the vein lies under its inner third.

**The Mortality of Cancer.**—Mr. Roger Williams[5] has compiled two tables which illustrate respectively the increase in the prevalence of cancer and the relative increase among males and females. The increase is not in particular parts of the body, but in all parts, and has not considerably altered the proportionate localisation ratios. In 1840 the proportion of deaths from cancer to population was 1 to 5,646; in 1896 it was 1 to 1,306; and the proportions to total deaths were respectively 1 to 129 and 1 to 22. The increase in cancer is far greater in proportion than the increase of population, and has been so steady and regular that it cannot be attributed to improved diagnosis or other casual error. The increase in mortality in males is nearly twice that in females. The writer believes that the great increase in the consumption of food, especially of meat, is one etiological factor of this increased cancer mortality. Another factor, he believes, is the greater urbanisation of the population, especially of males. Roswell Park[6] finds evidence of a similar increase of cancer mortality in the returns of the New York State Board of Health. These returns show that in 1895 the total number of deaths from cancer was double that in 1885.

[1] Birmingham Med. Review, Aug., 1898. [2] Brit. Med. Jour., June, 1898. [3] Johns Hopkins Hosp. Bull., July, 1898. [4] Brit. Med. Jour., April, 1898. [5] Lancet, Aug., 1898. [6] Amer. Jour. Med. Sci., May, 1898.

